# Proteasome inhibitors, including curcumin, improve pancreatic β-cell function and insulin sensitivity in diabetic mice

**DOI:** 10.1038/nutd.2016.13

**Published:** 2016-04-25

**Authors:** S Weisberg, R Leibel, D V Tortoriello

**Affiliations:** 1 Naomi Berrie Diabetes Center, Columbia University, New York, NY, USA; 2 Sher Institute for Reproductive Medicine, New York, NY, USA

## Abstract

**Background:**

Type 2 diabetes stems from obesity-associated insulin resistance, and in the genetically susceptible, concomitant pancreatic β-cell failure can occur, which further exacerbates hyperglycemia. Recent work by our group and others has shown that the natural polyphenol curcumin attenuates the development of insulin resistance and hyperglycemia in mouse models of hyperinsulinemic or compensated type 2 diabetes. Although several potential downstream molecular targets of curcumin exist, it is now recognized to be a direct inhibitor of proteasome activity. We now show that curcumin also prevents β-cell failure in a mouse model of uncompensated obesity-related insulin resistance (Lepr^db/db^ on the Kaliss background).

**Results:**

In this instance, dietary supplementation with curcumin prevented hyperglycemia, increased insulin production and lean body mass, and prolonged lifespan. In addition, we show that short-term *in vivo* treatment with low dosages of two molecularly distinct proteasome inhibitors celastrol and epoxomicin reverse hyperglycemia in mice with β-cell failure by increasing insulin production and insulin sensitivity.

**Conclusions:**

These studies suggest that proteasome inhibitors may prove useful for patients with diabetes by improving both β-cell function and relieving insulin resistance.

## Introduction

Diabetes is a disease in which the body does not sufficiently produce and/or respond to insulin, a pancreatic endocrine hormone crucial for maintaining glucose homeostasis. Insulin deficiency leads to hyperglycemia, which, if uncontrolled, will acutely cause life-threatening ketoacidosis and in the long-term foster vascular disease leading to potentially devastating end-organ failure. There are nearly 21 million Americans, or 7% of the population, who have diabetes, an epidemic mainly attributable to the marked increase in the incidence of obesity in this country.^1, 2, 3, 4^

Obesity is associated with enhanced adipocyte death and infiltration of macrophages into white adipose tissue.^5, 6^ Many inflammation-provoking 'adipokines', such as PAI-1 (plasminogen activator-1) and MCP-1 (monocyte chemoattractant protein-1), and macrophage-specific genes, including tumor necrosis factor-α and interleukin-6, are significantly upregulated in the white adipose tissue of obese subjects.^7, 8^ Within liver tissue, obesity and inflammation are linked by activation of the nuclear factor-κB (NF-κB) transcription factor. Genetic studies in mice have shown that interruption of NF-κB activity in hepatocytes and macrophages attenuates the development of insulin resistance in the setting of obesity.^9^ Thus, the development and progression of diabetes in obese individuals stems, in part, from these proinflammatory alterations that limit the response to insulin.

In the early stages of insulin resistance, hyperglycemia is usually averted due to pancreatic islet cell hyperplasia and hyperinsulinemia.^10^ The duration of this hyperinsulinemic, normoglycemic phase depends on both the severity of the insulin resistance and individual genetic factors. In patients with prolonged or severe insulin resistance, however, the metabolic stress imposed upon their β-cells often initiates their apoptotic death, necessitating exogenous insulin administration.

For most patients with diabetes, exercise and dietary management will prove insufficient to maintain euglycemia, and roughly 80% at some point will require either oral hypoglycemic agents and/or insulin injections for satisfactory blood glucose control. These drugs can be expensive and can induce unpleasant side effects or harmful toxicities.^11, 12^ Given the staggering prevalence and expense of treating diabetes and its associated complications, it is imperative to investigate alternative and/or complementary treatments that would ideally be safe, effective, inexpensive and readily available.

The dried ground rhizome of the perennial herb turmeric (*Curcuma longa*) is both a popular dietary spice as well as an integral part of the ancient Hindu medicinal system called Ayurveda.^13, 14^ Curcumin (diferuloylmethane) comprises 2–8% of most turmeric preparations and is generally regarded as its most active component. Commercial grade curcumin comes as a 95% standardized extract and contains the curcuminoids curcumin (~80%), desmethoxycurcumin (~10–20%) and bisdesmethoxycurcumin (<5%). Studies *in vitro* as well as in preclinical animal models of disease have shown that curcumin inhibits carcinogenesis^15, 16, 17, 18, 19, 20, 21, 22, 23, 24^ and inflammation.^13, 25, 26, 27, 28, 29^ In addition, we have recently demonstrated that dietary curcumin treatment attenuates insulin resistance and hyperglycemia in high-fat diet fed and genetically obese leptin-deficient C57BL/6 Lep^ob/ob^ mice by decreasing inflammation in adipose tissue and liver.^30^

Multiple different mechanisms, including NF-κB inhibition,^17, 31^ peroxisome proliferator-activated receptor-γ agonism,^32, 33^ cyclooxygenase-2 pathway inhibition^34, 35, 36, 37, 38^ and inhibition of the c-Jun N-terminal kinase signaling pathway,^39, 40^ have been implicated in curcumin's pleiotropic beneficial effects. Most recently, however, it has been established that curcumin is a *direct* inhibitor of the proteolytic core of the mammalian 26 S proteasome complex,^41, 42, 43, 44^ and evidence suggests that it is this ability that may, in fact, underlie its multiple downstream effects.^31, 44, 45, 46, 47, 48, 49, 50, 51, 52^

The proteasome works in concert with a tagging protein, ubiquitin, to create the ubiquitin-proteasome pathway, the major proteolytic pathway of eukaryotes. It controls the intracellular levels of a wide range of proteins, including NF-κB and others involved in the control of the cell cycle, transcriptional activation,^53, 54, 55, 56, 57^ apoptosis^43, 58, 59^ and cell signaling.^59, 60, 61, 62^ We postulated that proteasome inhibition may be contributing to the antidiabetogenic effects that we have shown curcumin to exert.

In this series of studies, we demonstrate that curcumin treatment enhances β-cell function and proliferation both *in vitro* and *in vivo*, and we implicate its inhibitory actions on the proteasome in its therapeutic activities. To model β-cell failure in the setting of insulin-resistant obesity, we used obese leptin receptor-deficient mice Lepr^db/db^ on the Kaliss (Ks) background. Unlike obese mice on the C57BL/6 background, the Ks Lepr^db/db^ mice are highly susceptible to β-cell failure in the setting of insulin resistance, and they develop early insulin depletion, severe hyperglycemia, ketoacidosis and early death. We found that feeding these animals a diet supplemented with curcumin markedly attenuated the development of hyperglycemia, increased circulating insulin levels, prevented β-cell loss and lengthened the lifespan.

In addition, short-term treatment of Ks Lepr^db/db^ mice with two structurally distinct proteasome inhibitors, epoxomicin and celastrol, significantly reduced hyperglycemia by concomitantly increasing insulin sensitivity and increasing insulin production. Taken together, these data suggest that one mechanism by which dietary supplementation with curcumin treats diabetes is by inhibiting the proteasome, a mechanism heretofore not described to possess antidiabetogenic properties.

## Materials and methods

### Experimental animals

*Dietary-induced obese C57BL/6J mice*: After 2–3 months, inbred *male* C57BL/6J wild-type mice will become obese and moderately diabetic if fed a high-fat diet (35% fat by weight). C57BL/6J *ob/ob* mice possess a spontaneous knockout mutation of the leptin gene that produces hyperphagia, decreased metabolic rate, severe obesity and moderate diabetes, which is eventually compensated for by pancreatic β-cell hyperplasia and hyperinsulinemia. C57BL/Ks *db/db* mice possess a spontaneous knockout mutation of the leptin receptor, which generates a phenotype initially very similar to that of the ob/ob mice. However, the loss of leptin effect on the C57BL/Ks background is not compensated for β-cell hyperplasia and hyperinsulinemia. At a very young age these mice become severely obese, hyperglycemic, hyperphagic and polydipsic. As they mature, they start to lose weight, develop nephropathy and ultimately die around age 40 weeks from diabetic complications.

All mice were obtained from the Jackson Laboratory (Bar Harbor, ME, USA). Mice were housed five mice per cage and maintained on a 12:12 light–dark cycle with a*d libitum* access to food and water. In the dietary curcumin experiments, the male Lep^ob/ob^ and Lepr^db/db^ mice were received at age 8–10 weeks and then randomized to receive a standardized 4% fat by weight meal diet (D12450B-I; Research Diets, New Brunswick, NJ, USA) containing either a 3% by weight admixture of curcumin or no additive. Assuming that each mouse weighed on average 40 g and consumed 2 g of food per day, their daily curcumin intake would be equivalent to 60 mg per day or 1.5 g kg^−1^ per day. The wild-type mice were obtained at age 3–5 weeks and randomized to receive either a standardized 4% fat by weight diet or, in an attempt to induce obesity, a high-fat diet containing 35% fat by weight (D12492-I; Research Diets). At the age of 20 weeks, these wild-type mice were further randomized with regard to the addition of a 3% by weight admixture of curcumin or no additive to their predesignated diet. Curcumin admixture dosage was based on our previous work with diabetic mice.^30^ Owing to obvious differences in food being consumed, blind analysis of mice experimental variables was not feasible.

The curcumin used was Curcumin C3 Complex (Sabinsa Corporation, Newark, NJ, USA), which is a 95% standardized curcumin extract. All mice remained on their assigned diet until being killed by CO_2_ asphyxiation at the age of ~28 weeks. All protocols were approved by the Columbia University Institutional Animal Care and Use Committee.

### Body composition analysis

The fat and lean tissue masses were calculated from living, non-anesthetized mice using a Bruker nuclear magnetic resonance machine (Bruker BioSpin Corp., Billerica, MA, USA). Pelleted food was weighed daily to assess total grams of food consumed per cage per day and then divided by 5 to estimate average daily food consumed per mouse.

### Glucose/HbA1c testing

Tail tip blood was collected from live mice to ascertain glucose and hemoglobin A1c (HbA1c) levels. Approximately 5 μl was used to assess blood glucose levels using a Freestyle Flash glucometer (Abbott, Abbott Park, IL, USA) and 25 μl was used to assess HbA1c using a Bayer DCA 2000+ analyzer (Siemens, Tarrytown, NY, USA).

### Insulin tolerance test

After fasting mice for 6 h to stabilize dietary glucose and insulin levels, ~25  μl tail tip blood was collected to ascertain fasting glucose and insulin levels. Mice then received 1.5 U kg^−1^ of regular insulin (Lilly, Indianapolis, IN, USA) by intraperitoneal injection. Sample tail tip blood glucose levels were assessed by glucometer at 15, 30, 45, 60 and 120 min.

### Histology

Pancreata were removed from mice at the end of treatment period and were fixed overnight at room temperature in zinc-formalin fixative ('Z-Fix' Anatech Ltd, Battle Creek, MI, USA) and then embedded in paraffin. Tissue was sliced into 5-μm sections cut at 50-μm intervals and then mounted on charged glass slides, deparaffinized in xylene and then stained. Slides were stained with polyclonal antibodies specific to insulin (sc-9168; Santa Cruz Biotechnology, Santa Cruz, CA, USA) and the proliferative marker Ki-67 (sc-7846; Santa Cruz Biotechnology).

### Short-term effect of proteasome inhibitors on murine islet cell transcription

Approximately 12-week-old male C57BL/Ks Lepr^db/db^ mice were given a single 100 μl intraperitoneal injection of vehicle (10% dimethyl sulfoxide, 70% 3:1 cremophor/ethanol and 20% phosphate-buffered saline), or vehicle with added curcumin (3 mg kg^−1^), celastrol (3 mg kg^−1^) (Calbiochem, Darmstadt, Germany) or epoxomicin (0.1 mg kg^−1^) (Calbiochem). Dosages of celastrol and epoxomicin were based on previous work in mice using these compounds.^63, 64^ After 24 h, they were killed by cervical dislocation, and collagenase (1.0 mg ml^−1^ in Hanks' balanced salt solution) was then injected into the pancreatic duct (5.0 ml per mouse). The distended pancreata were then incubated in a shaking water bath at 37 °C in 1.0 mg ml^−1^ collagenase in Hanks' balanced salt solution for 10 min, and the islets were recovered using Histopaque gradient solutions (3 ml of 1.119 g l^−1^, 3 ml of 1.083 g l^−1^ and 3 ml of 1.077 g l^−1^). After centrifugation for 20 min at 2500 *g*, islets were removed from the top layer and washed two times with RPMI-1640. *N*=5 per group.

### Quantitative real-time PCR

Total RNA was extracted from freshly removed pancreatic islet cells, 100 mg of flash-frozen mouse adipose or liver tissue or from freshly scraped cultured Ins-1 cells, using a commercially available acid-phenol reagent (TRIzol; Invitrogen, Carlsbad, CA, USA). First-strand cDNA was synthesized using SuperScript III reverse transcriptase and random hexamer primers as described in the manufacturer's protocol (Invitrogen). Samples of cDNA were diluted 1:25 in nuclease-free water (Qiagen Inc., Hilden, Germany). Samples from each cDNA pool were diluted 1:10, 1:30, 1:90 and 1:270 to create a standard curve for calculation of relative gene expression levels. PCR amplification mixtures (20 μl) contained 10 μl of 2x PCR SYBR Green I QuantiTect Master Mix (Qiagen Inc.), 0.4 μl of a mixture of 25 μm reverse and forward primers and 11.6 μl diluted cDNA template. Real-time quantitative PCR was carried out using the DNA Engine Opticon 2 Instrument (MJ Research Inc., Waltham, MA, USA) with the following cycling parameters: polymerase activation for 15 min at 95 °C and amplification for 40 cycles of 15 s at 94 °C, 10 s at 58 °C and 10 s at 72 °C. After amplification, melting-curve analysis confirmed the presence of a single amplicon in each instance.

For expression analysis, we used prevalidated real-time PCR primers (QuantiTect; Qiagen Inc.) for the genes analyzed including the 'housekeeping' control Rps3.

For each cDNA and standard curve sample, quantitative PCR reactions were performed to assay the expression of each internal control gene. To calculate the normalized relative expression levels of each gene assayed in each sample, we divided the relative gene expression value for that sample by the geometric mean of the relative expression values of the control genes. Separate analyses in which relative expression values were normalized with the relative expression values of each control gene yielded similar results.

### Cell viability assays

After treating Ins-1 cells with vehicle, curcumin, celastrol or epoxomicin, as described, 20 μl of a resazurin-containing reagent (CellTiter-Blue; Promega, Madison, WI, USA) was then added directly to each well. After a further incubation at 37 °C for 4 h, the fluorescence per well (560_Ex_/590_Em_) produced by the conversion of resazurin to resorufin, which is directly proportional to viable cell number, was quantified using a fluorescent plate reader.

### *In vitro* studies

*INS-1 cell studies*: INS-1 (832/13) β-cells (20) were plated at a density of ~2000 cells per well in 96-well trays in 100 μl of RPMI-1640 containing 11 mmol l^−1^ glucose, 10% (vol vol^−1^) fetal calf serum, 4 mmol l^−1^ l-glutamine, 100 IU ml^−1^ penicillin, 100 μg ml^−1^ streptomycin, 10 mmol l^−1^ HEPES, 1 mmol l^−1^ sodium pyruvate and 0.05 mmol l^−1^ β-mercaptoethanol. During the experiments, when achieving ~70% confluency, the Ins-1 cells were washed and given fresh media containing vehicle or the indicated concentrations of curcumin, celastrol or epoxomicin for 12 or 24 h. *N*=24 wells per group.

*3T3-L1 cell studies*: 3T3-L1 preadipocytes at 75% confluency were harvested from 25 mm^2^ flasks by trypsinization and then seeded in complete media (DMEM-high glucose, 10% fetal bovine serum, 1% penicillin) on 6-well plates at ~8000 cells per well. The cells were grown to confluency and then kept in this state for another 2 days to arrest cell division. They were then treated with adipocyte differentiation media (complete media containing dexamethasone 1 μm, 3-isobutyl-1-methylxanthine 0.5 mm and insulin 10 μg ml^−1^). After 4 days in this media, the media were carefully aspirated and the cells were then treated with adipocyte maturation media (complete media and insulin 10 μg ml^−1^ insulin), which was changed thereafter every 2 days for 10 days. At this point, the differentiated 3T3-L1 cells were subjected to treatment with adipocyte maturation media containing vehicle or varying concentrations of curcumin, celastrol or epoxomicin for 24 h. The cells were then harvested for RNA analyses.

All cell lines were kindly provided by the laboratory of Rudolph Leibel, MD, Columbia University Medical Center, New York, NY, USA.

### Hormone assays

After fasting for 6 h, mice were weighed and then had their blood glucose measured by glucometer (Glucometer Elite, Elkhart, IN, USA) using ~5 μl of tail tip blood. They were then killed by cervical dislocation. Their blood was then obtained by cardiac puncture, allowed to clot on ice for 3 h and then centrifuged at 10 000 *g* for 10 min. The sera were then transferred to clean vials for storage at −80 °C until the day of assay. Mouse serum insulin, leptin, MCP-1, adiponectin, tPAI-1 (tissue PAI-1) were quantified by ELISA (Linco Research Inc., St Charles, MO, USA). All inter- and intra-assay coefficients of variation were <10%.

### Statistics and definitions

Two-tailed Student's *t*-tests were used to compare serum analytes between C57BL/6J lean and obese experimental groups. Sample sizes of five to six mice were used in the treatment groups to generate means that were compared by two-tailed *t*-tests. To acquire at least 75% power, *α* was set at 0.05. A treatment effect of 50% difference in population means and a standard deviation of 30% was presumed. *P*<0.05 was considered statistically significant. All data were examined using the Sigmastat 2.0 software (Jandel Scientific, San Rafael, CA, USA).

## Results

### Effects of curcumin on glucose homeostasis in obese diabetic mice

**Figure 1:**
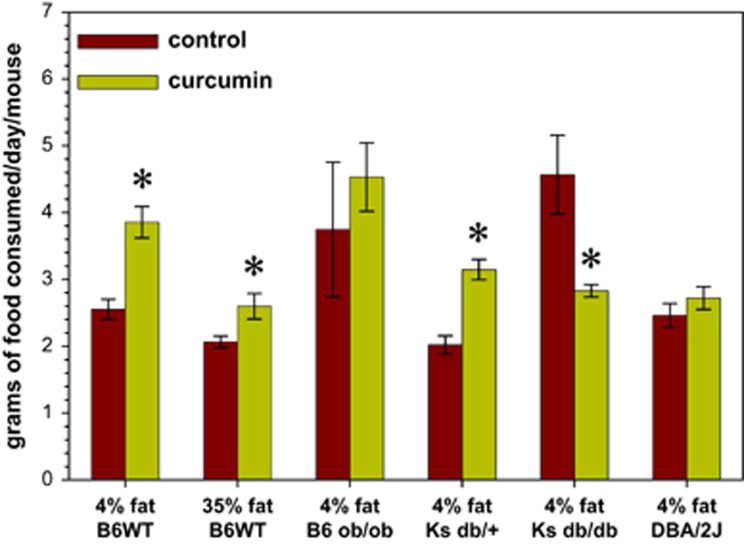
Most mice groups, lean and obese, consumed more food when curcumin (3%) was added to their diet. A notable exception was the C57BL/Ks Lepr^db/db^ mice, which actually consumed less food, likely due to an improvement in their glucose utilization. *N*=5 per group; *P<0.05 by two-tailed *t*-test.

To investigate the effects of curcumin in a mouse model of severe β-cell failure stemming from obesity and insulin resistance, we randomized 10-week-old Lepr^db/db^ male Ks mice to a 4% fat by weight diet (D12451; Research Diets, New Brunswick, NJ, USA) that was or was not supplemented with a 3% by weight 95% pure curcumin admixture (Sabinsa Corporation, Piscataway, NJ, USA). As a frame of reference, to assess the effect of dietary curcumin on normoglycemic mice with varying degrees of insulin sensitivity, we also subjected several other inbred mouse strains to dietary curcumin or not, namely wild-type male C57BL/6 J, wild-type male DBA/2 J, male Ks Lepr^db/+^ heterozygotes and male C57BL/6 J Lep^ob/ob^ homozygotes (Figure 1).

Similar to Lep^ob/ob^ mice on a C57BL/6 J (B6) background, the Lepr^db/db^ male Ks mice display hyperphagia secondary to a lack of leptin signaling and develop early-onset obesity.^65^ The addition of curcumin to the diet was associated with an increase in the grams of food consumed in the wild-type B6 mice as well as in the Ks Lepr^db/+^ heterozygotes (Figure 1). Interestingly, the total amount of food consumed by the Ks Lepr^db/db^ homozygotes was actually decreased by the addition of curcumin to their diet, an effect presumably due to their improved glucose utilization rather than any curcumin flavor aversion given the fact that the normoglycemic Ks Lepr^db/+^ heterozygotes behaved in an opposite manner (Figure 1). Mice with absolute leptin deficiency on the Ks background display early-onset insulin resistance with progressive β-cell failure that leads to severe insulin deficiency, wasting and death from ketoacidosis.^65^ Owing to genetic background differences, the male Lep^ob/ob^ mice on the C57BL6 background are able to rapidly compensate for their early diabetes through hyperinsulinemia^66^ stemming from pancreatic β-cell hyperplasia. It is not surprising therefore to see that the C57BL/6J Lep^ob/ob^ homozygotes showed no significant alteration in their food consumption (Figure 1).

**Figure 2:**
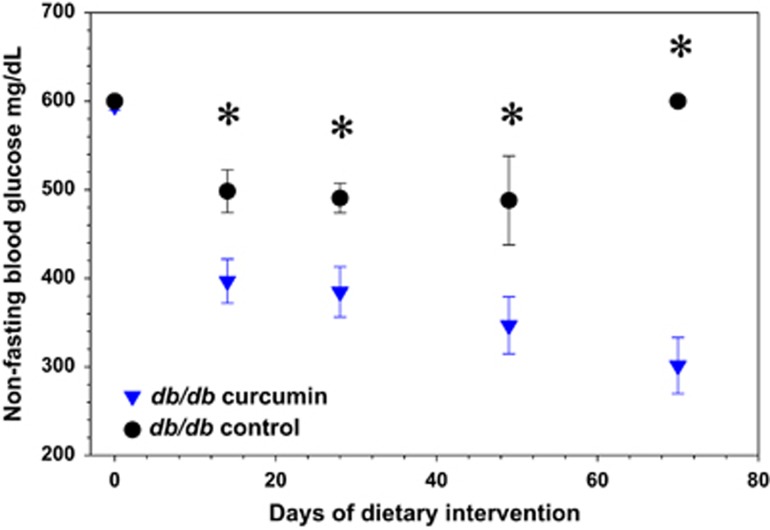
Dietary curcumin (3%) confers significant protection against hyperglycemia in male C57BL/Ks Lepr^db/db^ mice. *N*=5 per group; *P<0.05 by two-tailed *t*-test.

**Figure 3:**
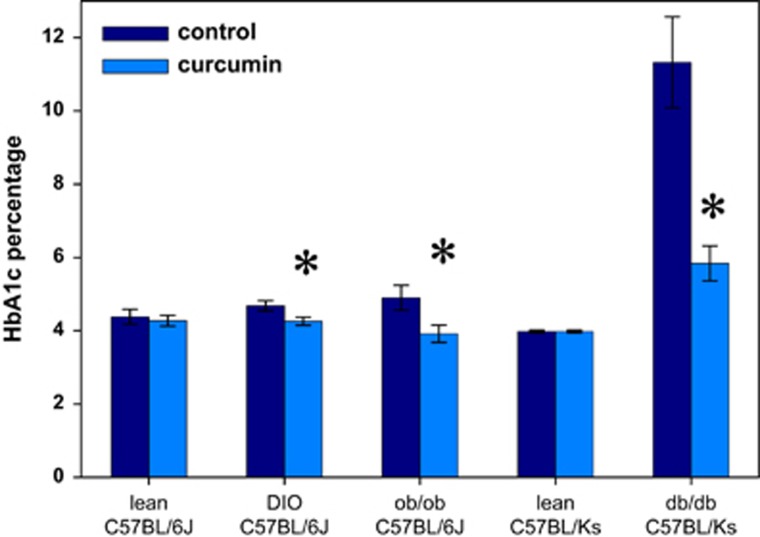
Dietary curcumin decreases HbA1c percentage in all mouse models of diabetes tested. Non-diabetic mice were not affected. *N*=6 per group. *P<0.05 by two-tailed *t*-test.

**Figure 4:**
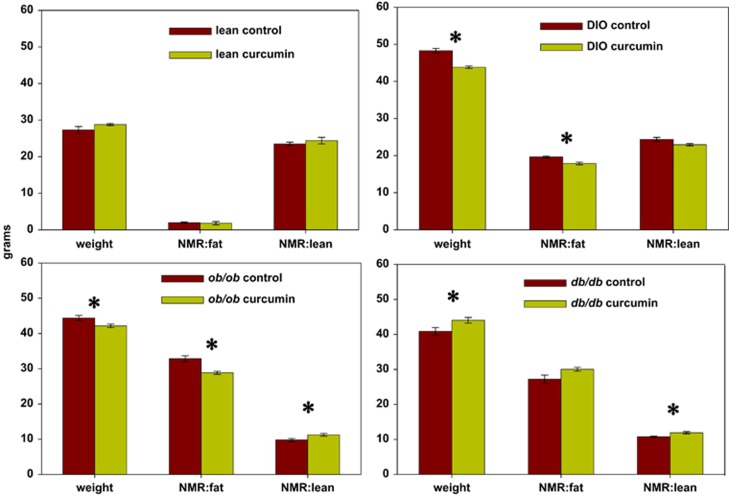
Effect of dietary curcumin on body composition. Nuclear magnetic resonance (NMR) spectroscopy revealed that dietary curcumin was associated with significantly diminished body weight and body fat percentage in dietary-induced obese (DIO) and C57BL/6 J Lep^ob/ob^ mouse groups, while it was associated with an increase in body weight and lean mass in the C57BL/Ks Lepr^db/db^ mice, likely by allowing them avoid the cachectic effects of poor glucose utilization and terminal ketoacidosis.

**Figure 5:**
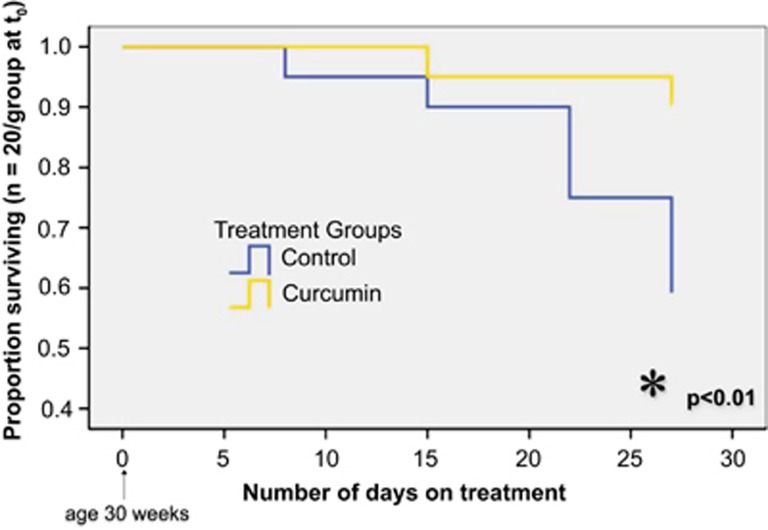
Dietary curcumin significantly improves survival in severely diabetic male C57BL/Ks Lepr^db/db^ mice. Beginning at 30 weeks of age, 40 C57BL/Ks Lep^db/db^ were randomized to either continue on their current diet or receive one supplemented by 3% curcumin. After 1 month of treatment, there was a statistically significant difference in the lifespans of the two groups demonstrating a beneficial effect of curcumin.

As early as 2 weeks following initiation of dietary curcumin, we noted a striking decline in the random glucose values of Lepr^db/db^ Ks mice in the curcumin-treated group compared with the controls, and this difference persisted over the 2.5-month treatment period (Figure 2). Accordingly, we also observed markedly decreased HbA1c percentages among all obese mice in the curcumin-treated group compared with controls (Figure 3). The most marked improvement, however, occurred in the Lepr^db/db^ Ks mice in which the curcumin treatment was associated with a >50% reduction in the HbA1c level (Figure 3). Curcumin had no effect on glucose levels or HbA1c percentages in lean mice and therefore does not appear to cause hypoglycemia. Unlike the Lep^ob/ob^ and dietary-induced obese mice, which actually lost weight and fat mass, as determined by nuclear magnetic resonance on curcumin treatment, the improvement in glucose metabolism mediated by curcumin in Lepr^db/db^ Ks mice allowed them to avoid the late-onset cachexia associated with diabetic ketosis and they weighed significantly more at the end of the treatment (Figure 4) compared with their control group, despite displaying decreased food intake (Figure 1). The increased body weight of the curcumin-fed Lepr^db/db^ Ks mice was attributable to an increase in their lean body mass as determined by nuclear magnetic resonance analysis (Figure 4). The salutary effects of curcumin on their glucose metabolism were sufficient to significantly extend the lifespan of curcumin-fed mice Lepr^db/db^ Ks compared with their controls (Figure 5).

### Effects of curcumin on β-cell function in obese diabetic mice

To investigate the mechanism by which curcumin prevents hyperglycemia in Lepr^db/db^ Ks mice, we measured fasting circulating insulin levels in lean Ks and obese Lepr^db/db^ Ks mice on control and curcumin-supplemented diets. We also examined β-cell proliferation and β-cell mass using immunohistochemical staining for insulin and the proliferation marker Ki-67 in pancreatic sections from these mice.

**Figure 6:**
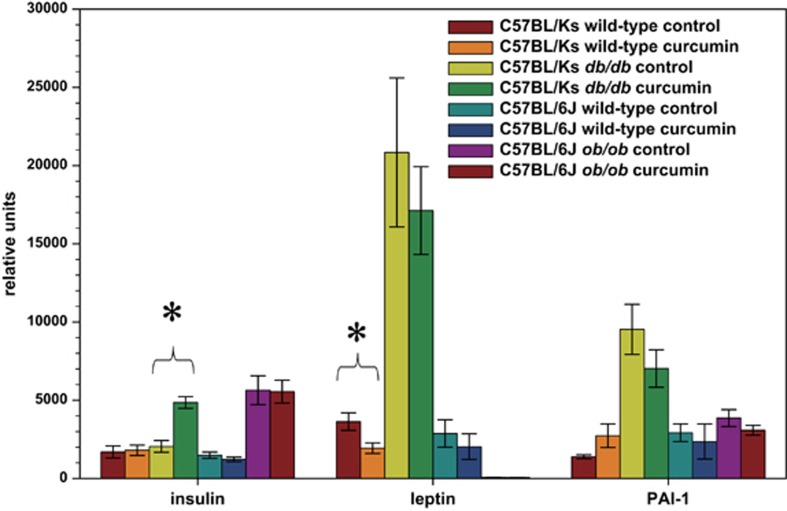
Dietary curcumin significantly increased serum insulin levels in male C57BL/Ks Lepr^db/db^ mice, while it was associated with a decrease in serum leptin levels in male C57BL/6J Lep^ob/ob^ mice. *N*=5 per group. *P<0.05 by two-tailed *t*-test.

Obese Lepr^db/db^ Ks mice on the control diet had similar circulating insulin levels as lean animals despite severe obesity and hyperglycemia (Figure 6)—a manifestation of severe β-cell failure. Accordingly, pancreatic sections of obese Ks Lepr^db/db^ mice on a regular diet showed no evidence of islet hypertrophy or β-cell proliferation compared with those of lean mice (Supplementary Figures 1d–f). In contrast, Lepr^db/db^ Ks mice on a curcumin-supplemented diet were able to generate a hyperinsulinemic response similar to that of the Lep^ob/ob^ B6 mice and had significantly increased circulating insulin levels compared with lean animals and their control-fed Lepr^db/db^ Ks mice (Figure 6). As expected, the obese Ks Lepr^db/db^ mice had the highest serum leptin and PAI-1 concentrations of all mice scrutinized, although curcumin did not significantly affect these levels (Figure 6).

Consistent with their elevated serum insulin levels, the pancreatic sections of Ks Lepr^db/db^ mice fed a curcumin-supplemented diet contained markedly enlarged islets with numerous Ki-67-positive β-cells (Supplementary Figures 1g–i) similar to those observed in obese Lep^ob/ob^ B6 animals with compensatory hyperinsulinemia (Supplementary Figures 1a–c). This suggests that curcumin may foster islet hyperplasia by increasing β-cell proliferation.

### Effects of proteasome inhibitors on glucose homeostasis and β-cell function in obese diabetic mice

Several molecular targets of curcumin have been described including NF-κB, as well as key regulators of the cell cycle and apoptosis. However, direct interactions between curcumin and these molecules have not been described. Recent work has demonstrated that curcumin binds to and directly inhibits the chymotrypsin-like activity of the 20S proteasome, the proteolytic core of the mammalian proteasome complex. We reasoned that if the therapeutic actions of curcumin were mediated to some degree by proteasome inhibition, then structurally distinct proteasome inhibitors would have similar therapeutic activities as curcumin. We selected two compounds for evaluation, celastrol and epoxomicin, both of which are potent and selective direct proteasome inhibitors with activities, similar to curcumin, that have been demonstrated both *in vivo* and in cell-free systems.^67, 68^ Owing to limitations imposed by the stability and cost of these compounds, they could not be administered over long periods of time as food admixtures.

We treated obese and hyperglycemic Ks Lepr^db/db^ mice with one dose of intraperitoneal celastrol (3 mg kg^−1^), epoxomicin (0.1 mg kg^−1^) or vehicle, and followed blood glucose values for 72 h postinjection. During this period, vehicle-treated mice were food entrained to the celastrol- and epoxomicin-treated mice to ensure that any changes in food intake induced by drug treatment would not influence blood glucose values. Between 12 and 40  postinjection, blood glucose levels in epoxomicin- (Supplementary Figure 2) and celastrol- (Supplementary Figure 3) treated mice remained significantly lower compared with those in vehicle-treated controls. Increased serum insulin levels were observed in celastrol- and epoxomicin-treated mice at time points corresponding to the peak hypoglycemic effects of these compounds (Supplementary Figure 4). In contrast, serum insulin values did not change significantly during the treatment period in vehicle-injected mice (Supplementary Figure 4).

### Effects of proteasome inhibitors on systemic insulin sensitivity

We have previously shown that long-term treatment with curcumin improves insulin sensitivity in obese mice, in part, by increasing adiponectin expression in adipose tissue and reducing expression of inflammatory mediators in adipose tissue. To determine if treatment with other proteasome inhibitors also increases insulin sensitivity in obese mice, we performed insulin tolerance tests on Ks Lepr^db/db^ mice 24 h after a single dose of celastrol (3 mg kg^−1^, intraperitoneally) or vehicle. Following an intraperitoneal bolus of insulin, celastrol-treated mice exhibited a significantly greater hypoglycemic response to insulin compared with vehicle-treated mice. Three days of daily celastrol intraperitoneal injections resulted in significantly decreased adipose tissue expression of *Ccl2* and significantly increased expression of adiponectin in the white adipose tissue of male Ks Lepr^db/db^ mice (Supplementary Figure 5).

### Effects of proteasome inhibitors on proliferative gene transcription

We used quantitative real-time PCR to identify transcriptional changes in islets that may contribute to the improved β-cell function induced by proteasome inhibition. RNA was extracted from islets purified from whole pancreas by collagenase digestion followed by gradient density centrifugation. Expression of Islet neogenesis-associated protein, which has been shown to directly increase β-cell mass, was significantly upregulated in islets from celastrol- and epoxomicin-treated mice compared with controls. Expression of the phosphatase and tensin homolog, a negative regulator of phosphoinositide kinase signaling, β-cell proliferation and survival (reference needed), and FOXO3a, a protein known to mitigate that compensatory adaptation of β-cell mass,^69^ was significantly downregulated in islets from celastrol- and epoxomicin-treated mice compared with controls (Supplementary Figure 6). Taken together, these gene expression changes may contribute to the beneficial effects of proteasome inhibitors on β-cell function.^70, 71^

### Effect of proteasome inhibitors on proliferation and insulin secretion in the β-cell line Ins-1

To investigate the ability of proteasome inhibition to directly improve β-cell function, we performed experiments using the rat insulinoma β-cell line Ins-1. We determined that the number of viable Ins-1 cells (CellTiter-Blue Cell Viability Assay; Promega) after 24 h in culture with varying concentrations of proteasome inhibitors was increased, except at the highest concentrations of celastrol and epoxomicin, which proved cytotoxic (Supplementary Figure 7a). When Ins-1 cells were cultured overnight in serum-free RPMI media containing varying concentrations of proteasome inhibitors, insulin secretion was significantly increased by proteasome inhibition, except at the highest concentrations of epoxomicin, which again proved cytotoxic (Supplementary Figure 7b).

### Effect of proteasome inhibitors on transcription of inflammatory and endoplasmic reticulum stress response proteins the adipocyte cell line 3T3-L1

Preadipocyte cells from the cell line 3T3-L1 were differentiated in culture until they achieved a state phenotypically compatible with mature adipocytes. They were then incubated overnight in varying concentrations of epoxomicin, curcumin or celastrol. Significant increases in the expression mRNA encoding the heat-shock proteins 70 and 90 were noted (Supplementary Figures 8a and b). Curcumin and epoxomicin were also noted to increase expression of the histone deacetylase activators sirt1 and sirt2.

## Discussion

The proteasome is thought to mediate the turnover of ~80% of cellular proteins. However, drugs that inhibit its core proteolytic activity have proven to be of clinical benefit and to have acceptable safety profiles. For example, the tripeptide proteasome inhibitor bortezomib is now routinely used in humans for relapsed and refractory multiple myeloma. Preclinical studies have shown promising antitumor activity and toxicity profiles of proteasome inhibitors in other animal models of human cancer.

Several mechanisms are postulated to underlie bortezomib's efficacy, but perhaps the most important is its ability to block NF-κB activation in a dose- and time-dependent manner by inhibiting the degradation of its binding partner and negative regulator, IκBα.^72^ Enhanced levels of IκBα lead to increased sequestration of the proinflammatory transcription factor NF-κB outside the nucleus. As a result, several NF-κB-dependent genes that foster carcinogenesis, angiogenesis, metastasis and severe inflammation are turned off, and NF-κB-dependent genes that foster inflammation are downregulated.

Obesity is associated with overproduction of multiple proinflammatory mediators that contribute to insulin resistance by activation of NF-κB.^73, 74^ Genetic or pharmacologic inhibition of this pathway with aspirin,^75^ adiponectin,^76^ thiazolidinediones^77^ or statins^78^ has been shown to ameliorate obesity-induced insulin resistance.^9, 79, 80, 81^ The hyperglycemia of diabetes, if unchecked, fosters further inflammation via oxidative damage. This phenomenon, referred to as 'glucose toxicity',^82^ is believed to be responsible for the progressive β-cell failure noted in poorly controlled type 2 diabetes.

In a previous report, we demonstrated that curcumin, a non-toxic compound purified from turmeric, prevented the development of obesity-induced insulin resistance and glucose intolerance primarily by attenuating hepatic and adipose tissue inflammation. The anti-inflammatory effect of curcumin in other systems and disease models is well established; however, its direct molecular target had not been identified until a recent report provided compelling evidence using both *in vivo* and cell-free systems that curcumin directly inhibits proteasome activity.

Here we have tested the hypothesis that curcumin's antidiabetes activity stems from proteasome inhibition by comparing its efficacy at treating type 2 diabetes in mice with that of two other structurally distinct proteasome inhibitors. We found that all three proteasome inhibitors ameliorate insulin resistance and β-cell failure.

Previously, we found that prolonged treatment with oral curcumin prevented insulin resistance and inflammation in obese mice that were not genetically susceptible to β-cell failure (C57BL/6 background). Here we tested the effects of curcumin therapy on obese mice with a strong genetic susceptibility to β-cell failure (Ks background). Our studies reveal that administration of proteasome inhibitors to these mice corrects their hyperglycemia, and appears to do so by increasing insulin sensitivity and by preserving β-cell function. In this system, the effects of curcumin on β-cell function were quite pronounced. After long-term dietary curcumin administration, the curcumin-treated male Lepr^db/db^ Ks mice had similar levels of islet hyperplasia and circulating insulin as male Lep^ob/ob^ C57BL/6 mice, indicating that curcumin almost completely overcame their genetic susceptibility to β-cell failure.

We also evaluated the effects of two structurally distinct proteasome inhibitors, celastrol and epoxomicin, on glucose levels, insulin sensitivity and β-cell function in obese diabetic mice. A single injection of these compounds raised circulating insulin levels within 24 h and substantially reduced hyperglycemia in these mice for nearly 48 h. Expression studies performed on the insulin-producing pancreatic islets of these mice showed that proteasome inhibition upregulated the expression of islet neogenesis-associated protein—a factor known to increase β-cell mass—and downregulated the expression of *Pten*—a negative regulator of β-cell growth and insulin secretion. In addition, short-term celastrol treatment improved systemic insulin responsiveness and increased the expression of adiponectin in adipose tissue.

We infer from these data that the common therapeutic activity of the structurally distinct compounds celastrol, curcumin and epoxomicin in obese and diabetic mice is related to their shared molecular target—the proteasome. It is also possible, however, that the antidiabetes activity of these three compounds is related to common off-target effects on molecules other than the proteasome. Further studies to evaluate the antidiabetes activity of a larger spectrum of specific proteasome inhibitors are needed to address this question.

Clinical trials in human patients using curcumin and celastrol to treat diseases such as arthritis and amyotrophic lateral sclerosis are currently underway. Although toxicities associated with celastrol are less well defined, curcumin has been shown to be non-toxic at very high dosages in humans.^83^ The data presented here suggest that curcumin, celastrol or other proteasome inhibitors warrant further evaluation for the treatment of type 2 diabetes in humans.
