# Supplementary figures and images for: Proteasome inhibitors, including curcumin, improve pancreatic β-cell function and insulin sensitivity in diabetic mice
Source: Nutr Diabetes. 2016 Apr 25;6(4):e205–. doi: 10.1038/nutd.2016.13 (PMC4855258; doi:10.1038/nutd.2016.13)

## Slide 1
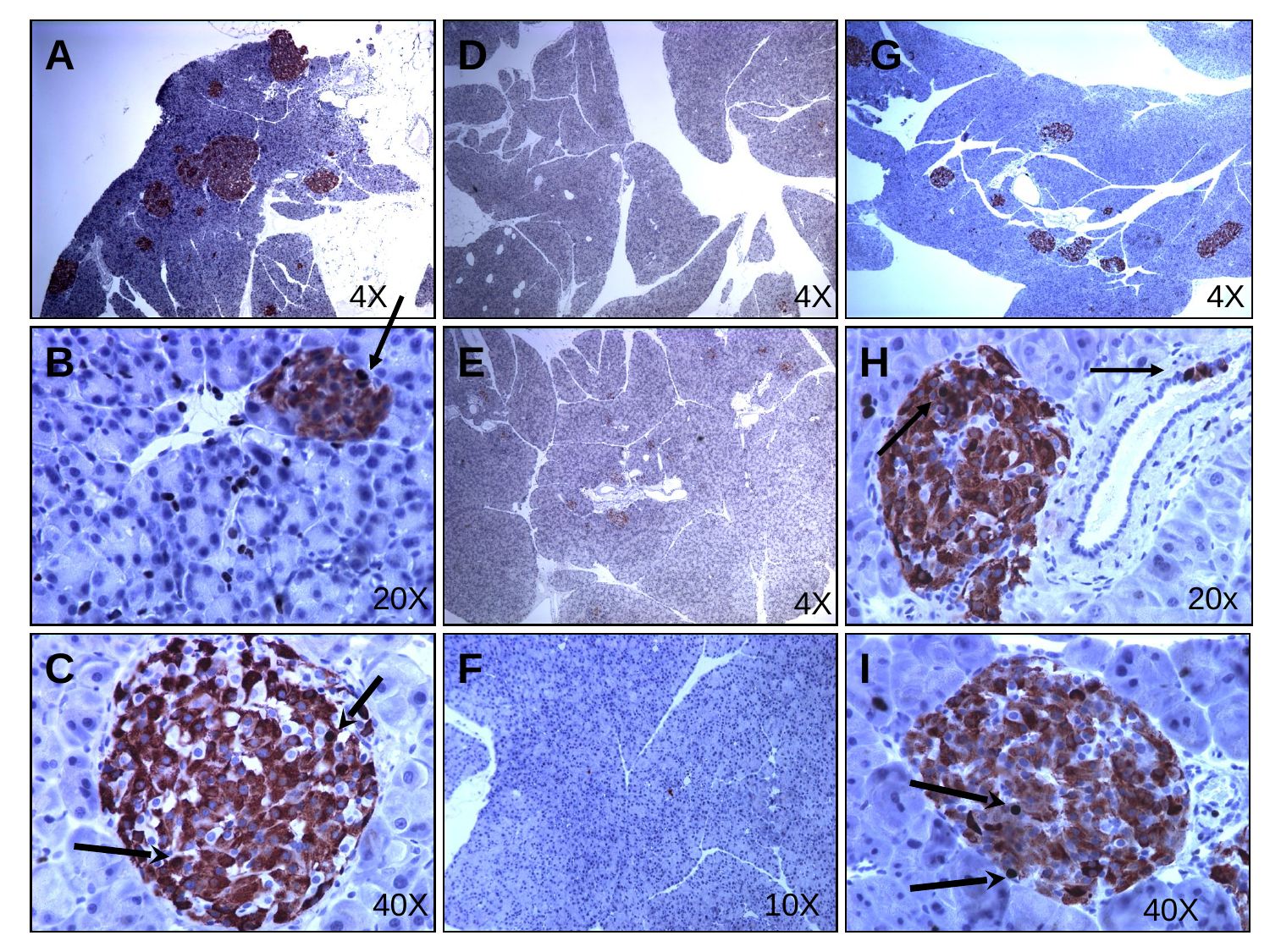

A
D
G
4X
4X
4X
B
E
H
20X
20x
4X
C
F
I
40X
10X
40X
Supplemental Figure 1

Supplement: Supplementary Figures 1 [file nutd201613x1.ppt]

## Slide 1
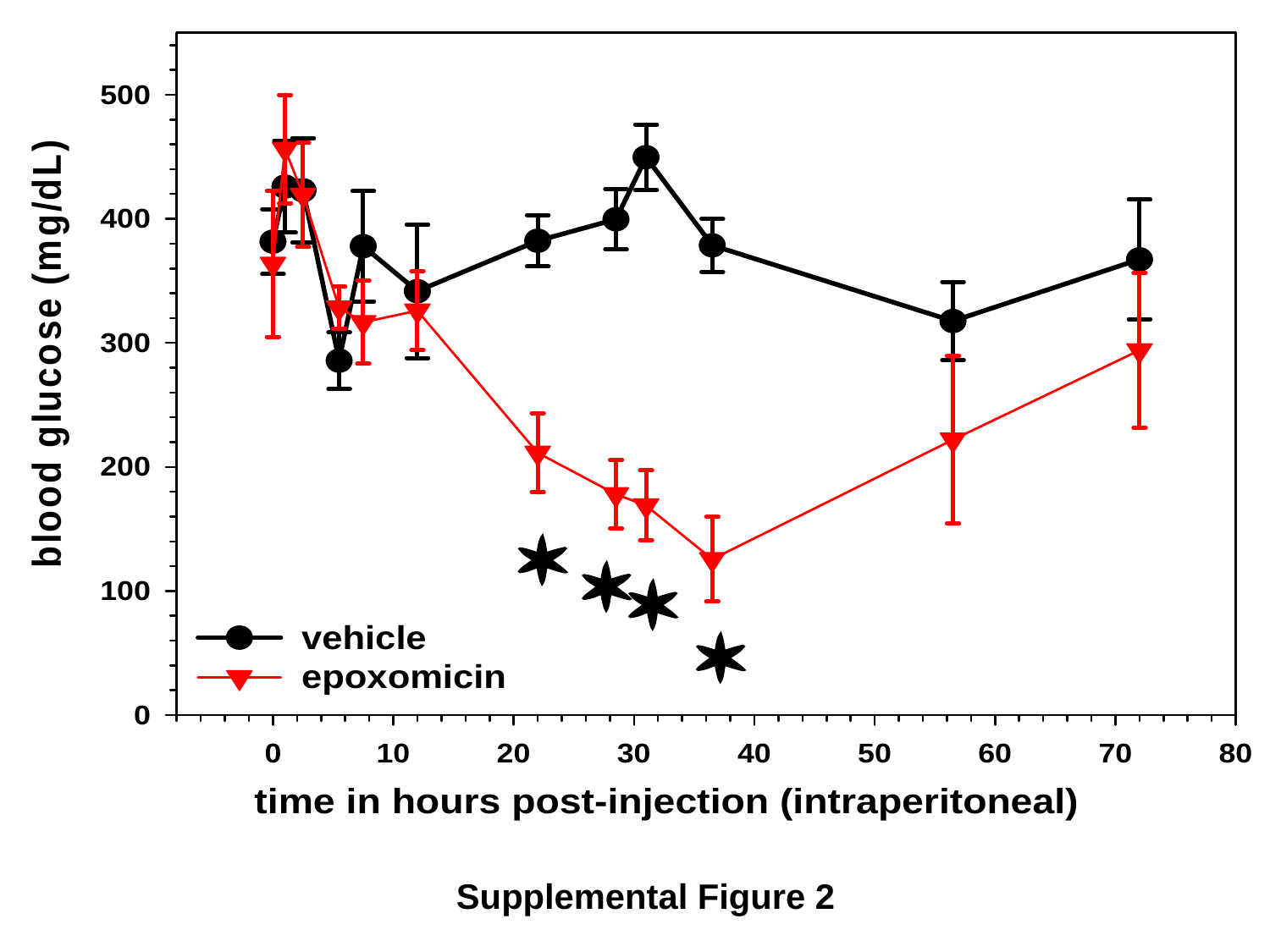

Supplemental Figure 2

Supplement: Supplementary Figures 2 [file nutd201613x2.ppt]

## Slide 1
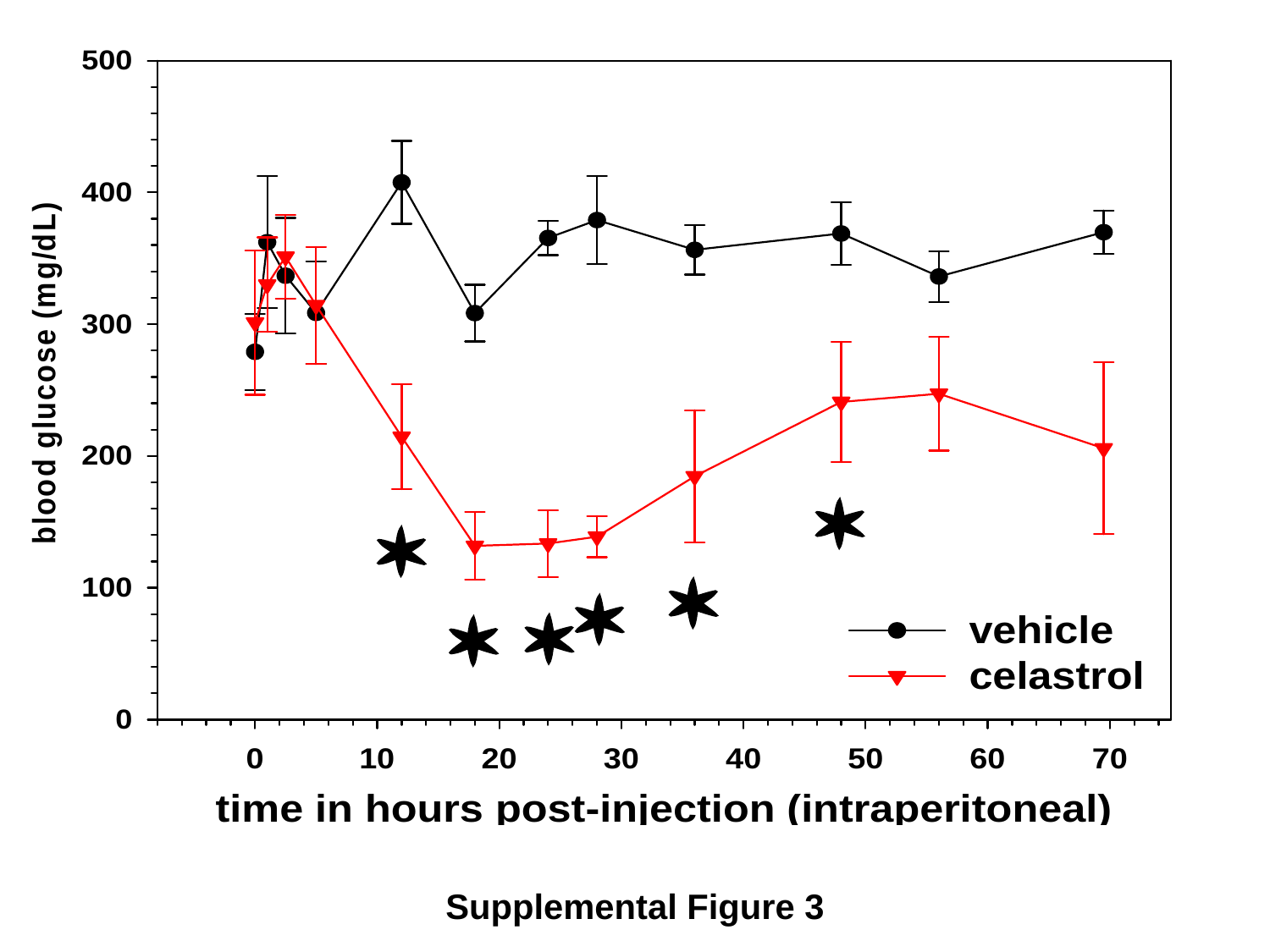

Supplemental Figure 3

Supplement: Supplementary Figures 3 [file nutd201613x3.ppt]

## Slide 1
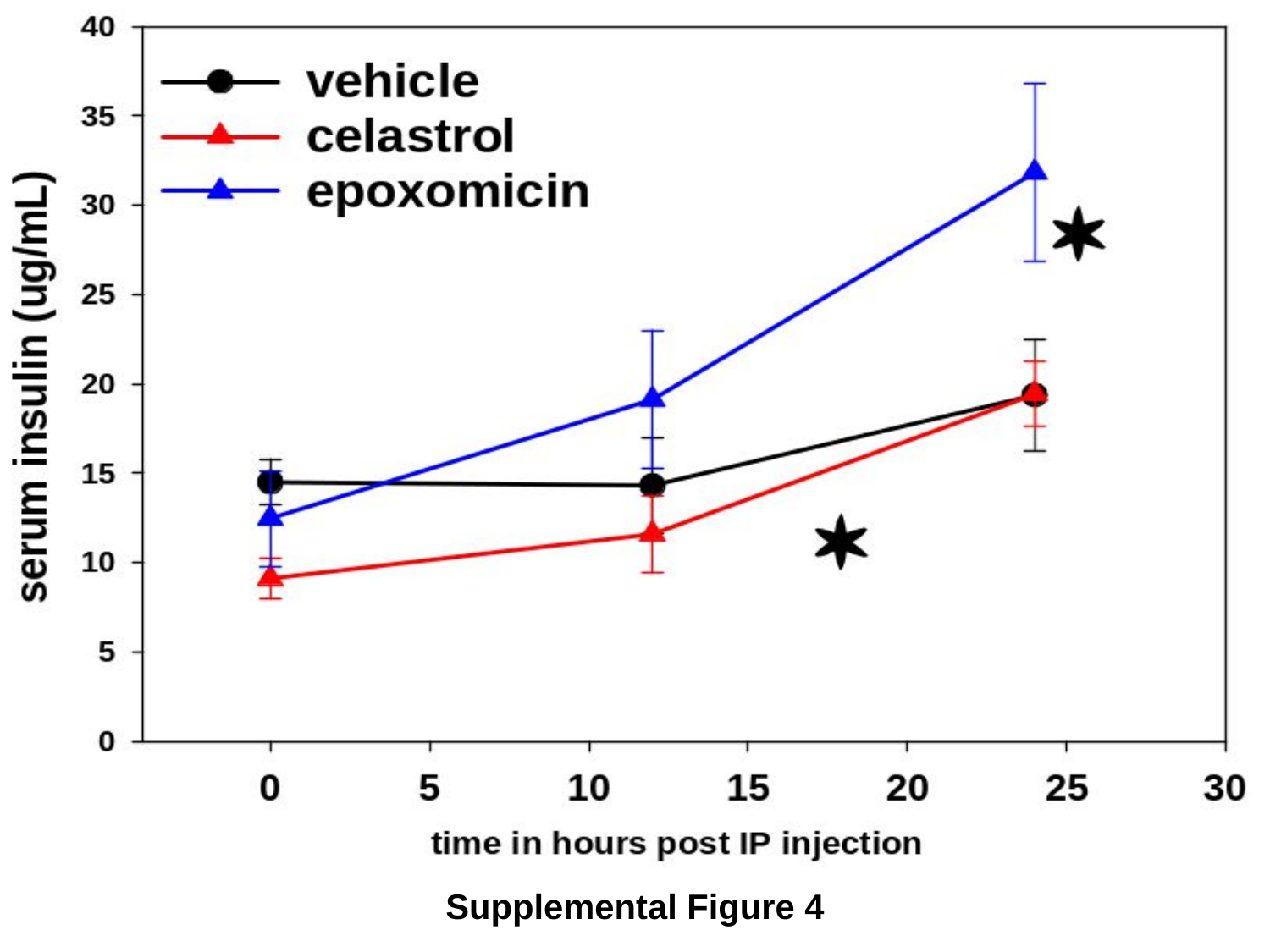

Supplemental Figure 4

Supplement: Supplementary Figures 4 [file nutd201613x4.ppt]

## Slide 1
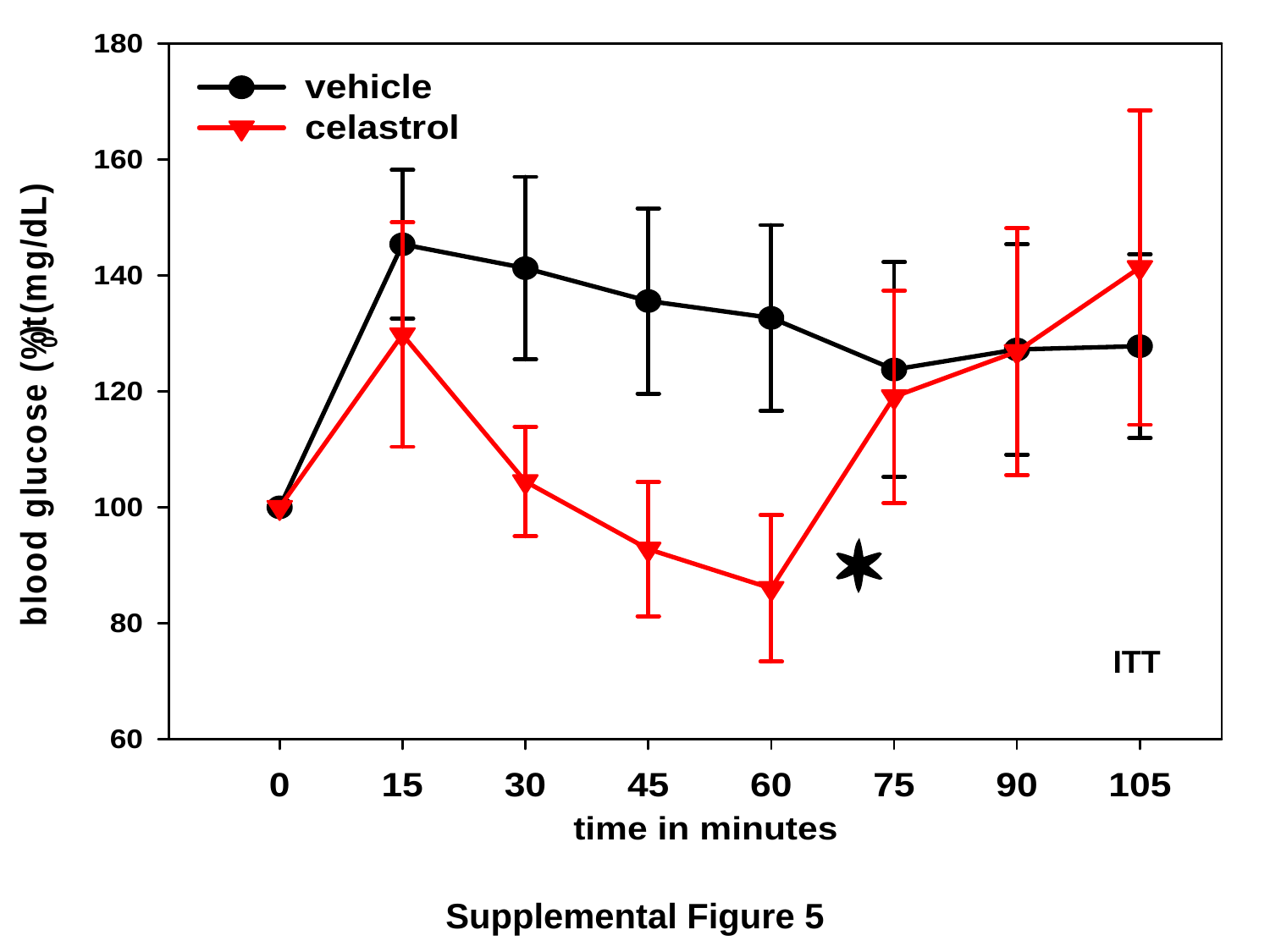


ITT
Supplemental Figure 5

Supplement: Supplementary Figures 5 [file nutd201613x5.ppt]

## Slide 1
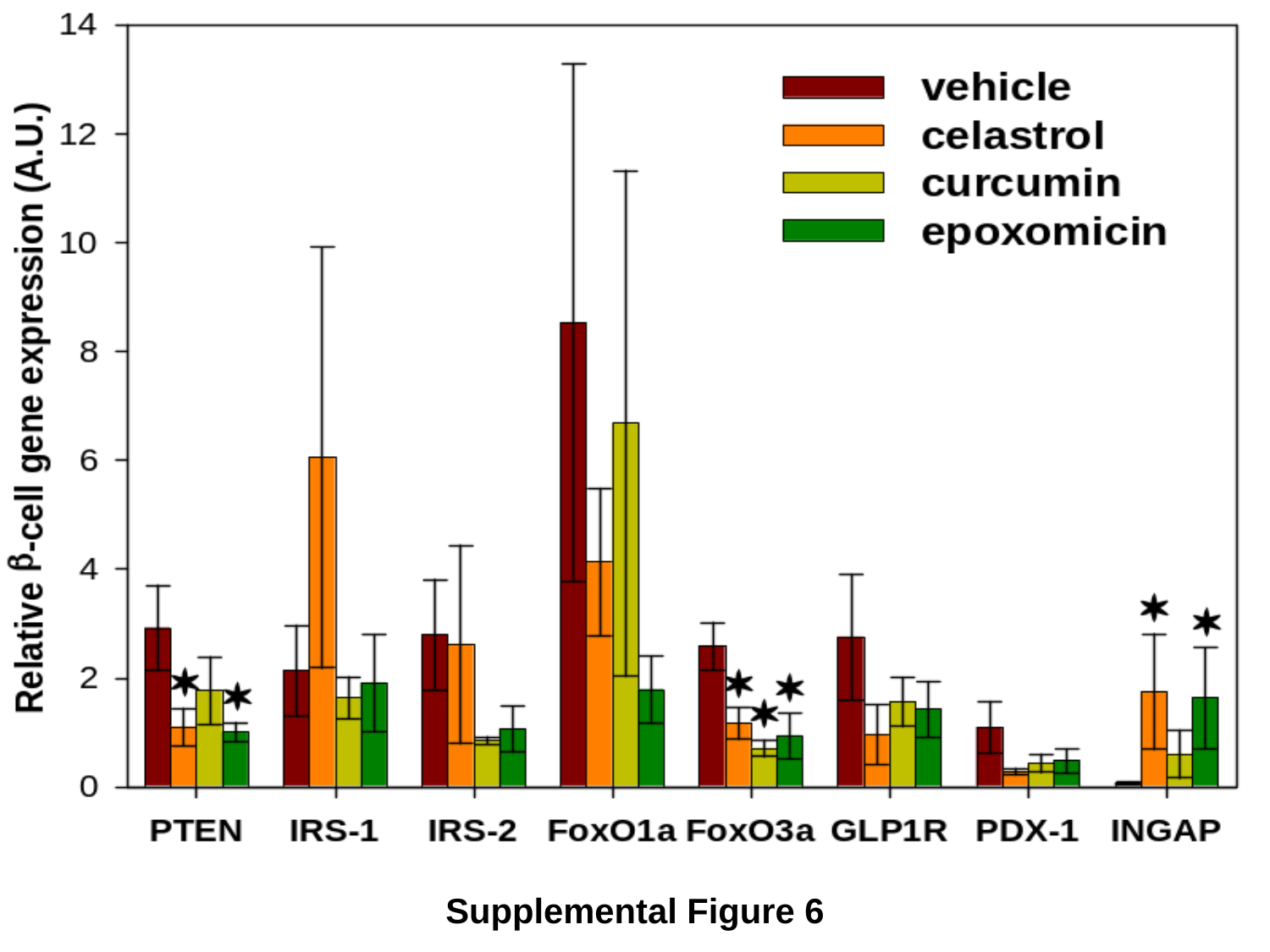

# Supplemental Figure 6

Supplement: Supplementary Figures 6 [file nutd201613x6.ppt]

## Slide 1
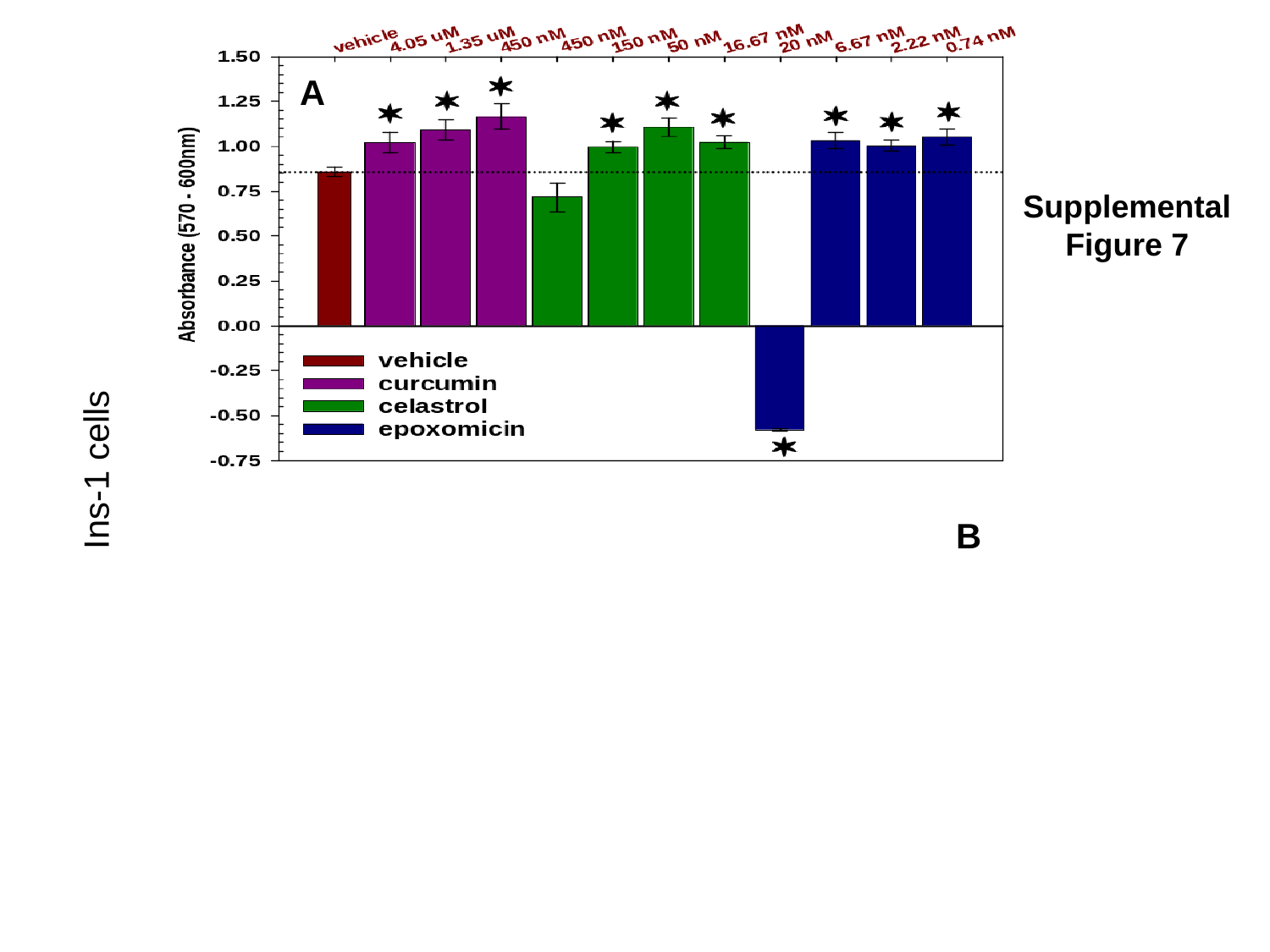

A
Supplemental Figure 7
Ins-1 cells
B

Supplement: Supplementary Figures 7 [file nutd201613x7.ppt]

## Slide 1
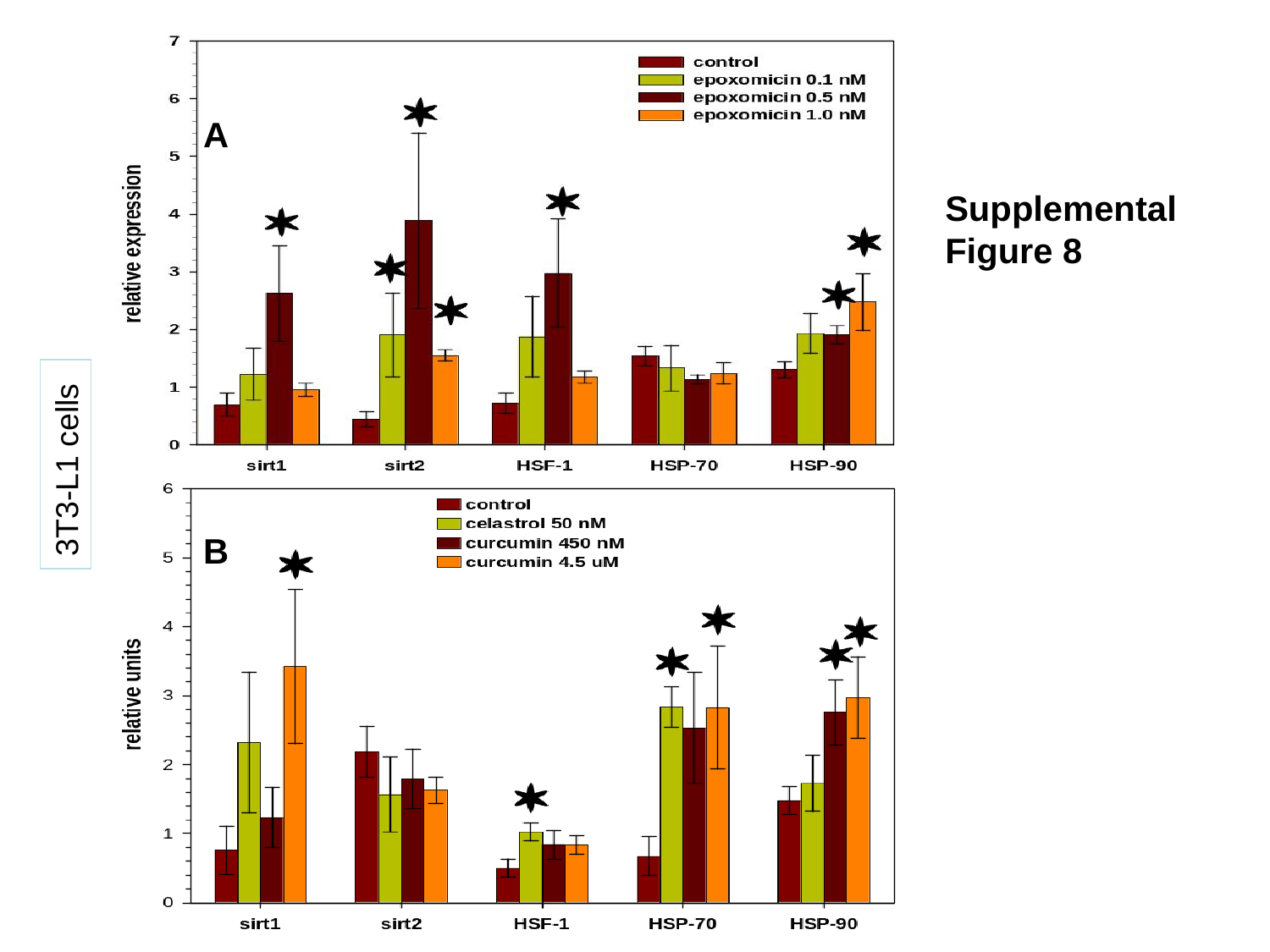

A
Supplemental
Figure 8
3T3-L1 cells
B

Supplement: Supplementary Figures 8 [file nutd201613x8.ppt]
