# Supplementary material for: Proteasome inhibitors, including curcumin, improve pancreatic β-cell function and insulin sensitivity in diabetic mice
Source: Nutr Diabetes. 2016 Apr 25;6(4):e205–. doi: 10.1038/nutd.2016.13 (PMC4855258; doi:10.1038/nutd.2016.13)
Supplement: Supplementary Figures Legends [file nutd201613x9.docx]

**Supplemental Figure 1.** By age 20 weeks, untreated C57BL/6J Lep^ob/ob^ mice (a-c) and curcumin-treated C57BL/Ks Lepr^db/db^ mice (g-i) manifest hyperplasia of the pancreatic islets. Untreated C57BL/Ks Lepr^db/db^ mice manifest islet depletion (d-f). Arrows point to nuclei positive for Ki67, a proliferation marker.

**Supplemental Figure 2.** A single intraperitoneal injection of epoxomicin significantly lowers blood glucose for nearly 2 days thereafter in male C57BL/Ks db/db mice as compared to a food-entrained control group. N=5 per group; *signifies p<0.05 by two-tailed t-test

**Supplemental Figure 3.** A single intraperitoneal injection of celastrol significantly lowers blood glucose for nearly 2 days thereafter in male C57BL/Ks Lepr^db/db^ as compared to a food-entrained control group. N=5 per group; *signifies p<0.05 by two-tailed t-test

**Supplemental Figure 4.** Intraperitoneal administration of a single dose of celastrol and epoxomicin significantly increases serum insulin in male C57BL/Ks Lepr^db/db^ mice after 24 hours. N = 6 per group; * signifies p<0.05 by two-tailed t-test

**Supplemental Figure 5.** Celastrol injections significantly lower AUC of ITT in male C57BL/Ks Lepr^db/db^ mice. n=5 per group; * signifies p<0.05

**Supplemental Figure 6.** PTEN and Foxo3a expression in pancreatic b-cells of male C57BL/Ks Lepr^db/db^ mice is significantly decreased 24 hours after a single intraperitoneal injection of proteasome inhibitors. INGAP expression is significantly increased. All values are mean ± SEM; N= 6 per group; * signifies p<0.05 by two-tailed t-test.

**Supplemental Figure 7.** A. The effect of proteasome inhibition on the rat β-cell line INS-1. All proteasome inhibitors were able to significantly increase viable cell number compared to vehicle after 24 hours. However, at their highest concentrations, celastrol and epoxomicin exerted negative effects on cell viability. B. Proteasome inhibitors fostered an increase in insulin secretion in Ins-1 cells after being in culture 12 hours. The highest concentrations of epoxomicin exerted a negative effect, likely due to cytotoxicity. All values are mean ± SD; n=3 replicate wells per group.

**Supplemental Figure 8.** A. Epoxomicin upregulates transcription of sirtuins and heat shock proteins 70 and 90 in differentiated 3T3-L1 adipocyte cells in vitro. B. Celastrol and curcumin upregulate transcription of sirtuins and heat shock proteins 70 and 90 in differentiated 3T3-L1 adipocyte cells *in vitro*.
